# Reporting involvement activities with children and young people in paediatric research: a framework analysis

**DOI:** 10.1186/s40900-023-00477-8

**Published:** 2023-07-31

**Authors:** Jennifer Preston, Giovanni Biglino, Victoria Harbottle, Emma Dalrymple, Helen Stalford, Michael W. Beresford

**Affiliations:** 1 Institute of Life Course and Medical Sciences, University of Liverpool, Liverpool, UK; 2 Bristol Medical School, University of Bristol, Bristol, UK; 3 Population Health Sciences Institute, University of Newcastle, Newcastle upon Tyne, UK; 4 Rehabilitation Department, Great North Children’s Hospital, Newcastle upon Tyne, UK; 5 Institute of Child Health, University College London, London, UK; 6 School of Social Justice and Law, University of Liverpool, Liverpool, UK; 7 Department of Paediatric Rheumatology, Alder Hey Children’s NHS Foundation Trust, Liverpool, UK

## Abstract

**Background:**

The active involvement of patients and the public in the design and delivery of health research has been increasingly encouraged, if not enforced. Knowledge of how this is realised in practice, especially where children and young people (CYP) are concerned, is limited, partly due to the low level of reporting of patient and public involvement (PPI) in general. The aim of this work was to assess how researchers funded by the National Institute for Health and Care Research (NIHR) report the involvement of CYP in the design and conduct of child health research to better understand the opportunities offered to CYP, and the realities of involvement in practice.

**Methods:**

A participation matrix, analysis framework and accompanying tools were adapted from existing frameworks, including a child-rights informed framework, the Guidance for Reporting Involvement of Patients and the Public Checklist Short Form (GRIPP2SF), and NIHR reporting expectations. Child-focused research reports were identified from the NIHR Journals Library, including any interventional or observational study involving CYP aged 0–< 24 years. In two co-design workshops with healthcare professionals and CYP, we tested and refined the participation matrix, analysis framework and accompanying tools.

**Results:**

Only thirty-two NIHR reports out of 169 (19%) were identified as relevant and included reporting of PPI with CYP. We identified significant variability in the way PPI with CYP was reported. Only 4/32 (12%) reports fully met NIHR (and GRIPP2SF) reporting criteria. Only 3/32 (9%) reports formally evaluated or self-reflected on PPI activities with CYP, whilst 15/32 (47%) provided minimal information about CYP involvement. The most common approach to involving CYP (23/32, 72%) was through the medium of existing groups or networks.

**Conclusion:**

Despite the NIHR’s commitment to increase the quality, transparency, and consistency of reporting PPI, the reporting of involvement with CYP remains sub-optimal. Neglecting to report key details of involvement methods and impacts deprives the research community of knowledge to advance the field of delivering ‘meaningful’ PPI with CYP. Practical guidance on how researchers can report the processes and outputs of CYP involvement more rigorously may help child health researchers to involve them more meaningfully. This research offers practical tools informed by CYP to aid the reporting process.

**Supplementary Information:**

The online version contains supplementary material available at 10.1186/s40900-023-00477-8.

## Abstract (plain language summary)

**Background:**

Children and young people can (and should) be involved in the design of child-health research. How this works in practice is limited due to the low-level reporting of involvement activities.

**Aim:**

We wanted to understand how researchers funded by the National Institute for Health and Care Research (NIHR) are reporting involvement activities with children and young people in child-health research.

**What we did and how we did it:**

A team of healthcare researchers (working in child-health research), a parent, and children and young people adapted existing tools to assess how the involvement of children and young people is reported in child-health research. We looked at reports in an online library to see how researchers are meeting the reporting guidelines set by the NIHR.

**Findings:**

Despite the reporting guidelines provided by the NIHR, only four out of thirty-two reports followed this. It is still unclear who is involved, why, and what outcomes and impact involvement has on the research process, on children and young people and/or on researchers. This is a missed opportunity to learn from previous experiences and to build on good practice. Further work is needed to understand why and how the NIHR, and other funders of health research collect such information, and what their plans are for sharing the findings with the wider research community.

**Supplementary Information:**

The online version contains supplementary material available at 10.1186/s40900-023-00477-8.

## Background

Active involvement of patients and the public in the design and delivery of health research, rather than as ‘subjects’ of research has been encouraged (if not required) for many years through policy [1, 2], regulations [3, 4], funders, [5, 6] and best practice guidance [7–9].

Patient and public involvement (PPI) encompasses initiatives to include patients, family members, carers and members of the public in developing and improving health services and medicines [10]. There are many definitions used to describe PPI interactions, which are often used interchangeably. These include, ‘*participation’* (when individuals take part in the actual research, [11] ‘*engagement’* (when research information is shared with the public, e.g. at research open days or on social media [11–13]), and ‘*involvement’* most commonly defined as *‘research being carried out ‘with’ or ‘by’ members of the public rather than ‘to’, ‘about’ or ‘for’ them’* (indicating a more active collaboration between patients, public and researchers) [11].

In recent years, there has been a growing call to include the voices of CYP in societal decision-making (including in healthcare and health research) in order to ensure that policies and programmes are more responsive and relevant to the concerns and needs of CYP [14, 15]. Meaningful involvement is considered to be a fundamental human right as articulated in the United Nations Convention on the Rights of the Child (UNCRC) [16]. The UNCRC is binding on the government and on public authorities at all levels in the UK and provides a strong ethical framework for planning and delivering meaningful involvement of CYP.

However, defining how meaningful PPI is realised in practice, especially where child and young patients and the public are concerned is limited [17–19], partly due to the low level of reporting PPI in general [20]. Recent attempts to synthesise what is known from the literature about PPI with CYP concluded that improvements need to be made to the evaluation and reporting of PPI with CYP, in order for researchers and funders to better understand the different levels and roles CYP have, and hence what works best for them, in different settings, and what impact their involvement has on the actual research itself and on those who get involved [21–23].Thus the resulting evidence base remains disjointed, and shared learning from previous experiences is lost, which potentially is a waste of resource that could otherwise be put to informing ‘meaningful’ involvement of CYP [21].

To address the issue of poor reporting of PPI, in April 2018 the National Institute for Health and Care Research (NIHR) advised authors of research it funds to refer to the Guidance for Reporting Involvement of Patients and the Public Checklist (GRIPP2) [24] to enhance the quality, transparency, and consistency of reporting PPI activities. The NIHR is one of the UK’s leading funding bodies that has provided vital strategic and infrastructure support to embed PPI across publicly funded research, creating an environment that views PPI as a crucial component of the research process [25]. It is one of the first health research funders to publish comprehensive accounts of its commissioned research within its own publicly and permanently available journals. The NIHR Journals Library^1^ comprises a suite of five open access peer-reviewed journals reporting results from several of its NIHR Programmes, which address a range of health research priorities. Reports published in the NIHR Journals Library provide a full account of the research project, including methods and a full description of the results. Further, in keeping with its role of providing a comprehensive archive of funded research, all reports in the library should include an explanation of how patients and the public have been involved in the study. Authors are encouraged to report faithfully on PPI activities undertaken, even if only to acknowledge the absence of it within the study. The NIHR reporting expectations require authors of reports to describe the following: if there was no PPI in the study to state this in the report, setting out why this was not thought appropriate or was not feasible; what form the PPI took and at what stages it occurred during the study; what impact PPI had during the study and how it was useful; if there was little/no impact of PPI during the study, to state it; and the way(s) PPI supported or will support dissemination of the results.

Furthermore, the GRIPP2 Checklist was introduced as a guide for authors. The Checklist was developed using the EQUATOR method [26] for developing reporting guidelines. The Checklist consists of two forms; a short form (SF) version referred to as GRIPP2-SF used primarily for studies where PPI is a secondary focus, and includes five items on aims, methods, results, outcomes, and critical perspective. The long form (LF) version referred to as GRIPP2-LF is aimed at studies where the main focus is PPI, and includes thirty-four items on aims, definitions, concepts and theory, methods, stages and nature of involvement, context, capture or measurement of impact, outcomes, economic assessment, and reflections.

As of the 1st of April 2022, it is mandatory for all reports to include a separate section on PPI as a sub-heading in the discussion section covering details of the PPI approach (or justification for no PPI), and what impact this had on all aspects of the study.

As a result of the reporting guideline changes within NIHR, we decided to explore how CYP’s involvement in the design and conduct of paediatric research (in any type of intervention, comparison, or outcome) is reported within the NIHR Journals Library. In this report we use the term ‘involvement activities’ to define the inclusion of CYP taking part in research advisory roles (as part of research advisory, focus or steering groups, etc.), advising on various or all aspects of study design and conduct.

## Aim

The aim was to examine in detail reports that are completed by researchers about involvement activities with CYP. Only sections of reports that described the involvement of CYP in these types of activities, not adult involvement (parents and other stakeholders) were included in the analysis. We wanted to explore:

1. Who is involved and how?
2. What are the reported opportunities offered to CYP, including levels of involvement in the different phases of the research process?
3. What are the reported outcomes and impacts of PPI with CYP?
4. Do CYP support the dissemination of research findings and how?
5. What are the reported challenges and facilitators to involvement?

Ultimately, we analysed reports published in the NIHR Journals Library in 2018 up to 28th February 2022 to build a picture of how NIHR-funded researchers are reporting PPI with CYP. We acknowledge that documentary evidence is not a proxy for actual PPI practice. It is not our intention to offer judgements about practice or individuals responsible for writing the reports.

## Methods

*Step 1*: Participation matrix and assessment tool.

**Fig. 1:**
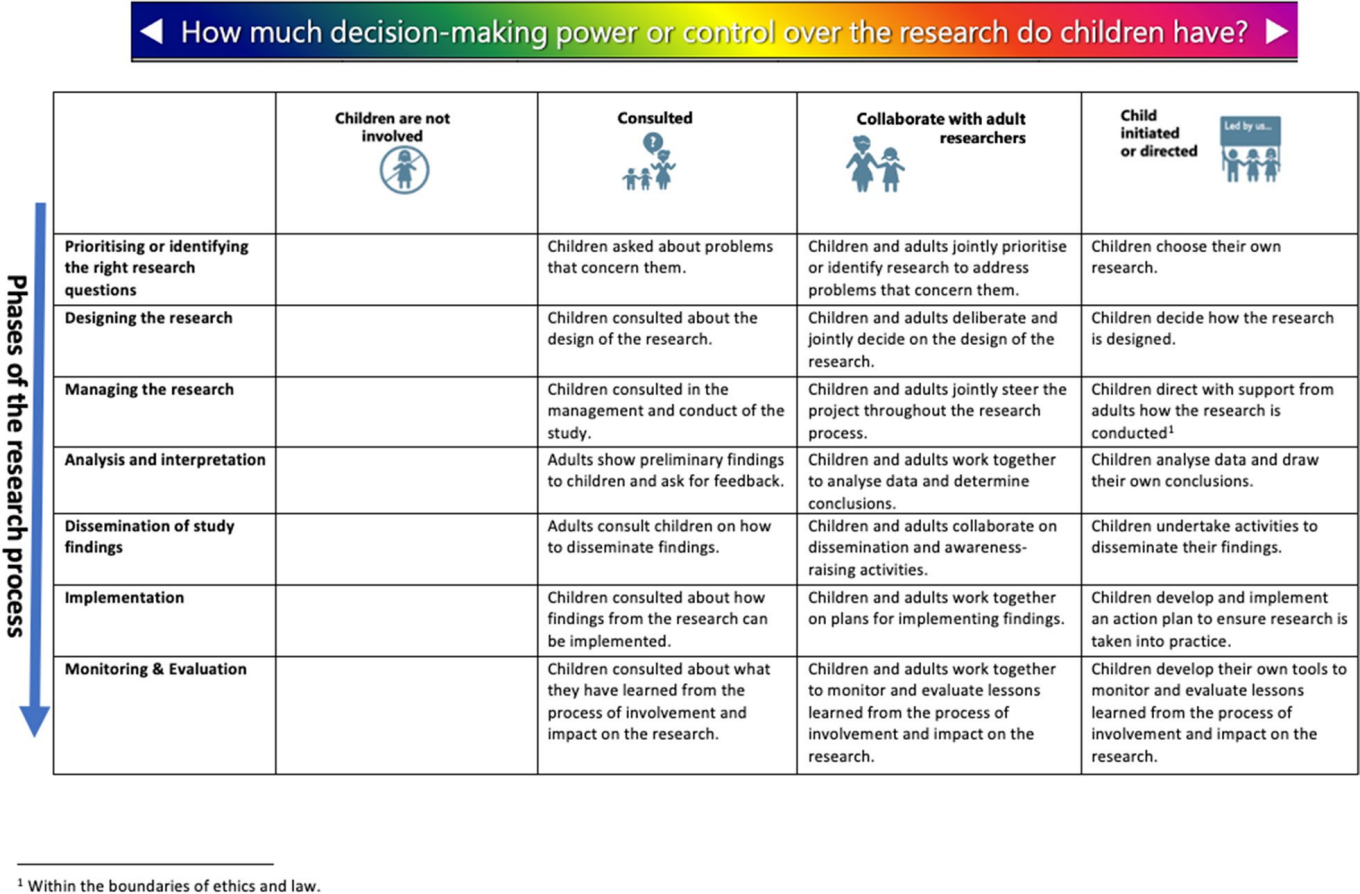
Participation Matrix

To assess the reported opportunities offered to CYP including the levels of involvement across the various phases of the research process, we adapted an existing Participation Matrix (hereon referred to as a matrix) originally developed by Lansdown [27] (See Fig. 1) to promote the participation rights of CYP. The matrix has predominantly been used in social science research/policy and practice. It was originally created to acknowledge that CYP can participate in activities, processes, and decision-making in three different ways: consultation, collaboration or child led. It was designed not to be hierarchical in nature but to be used to reflect different degrees of empowerment and influence that are all legitimate and appropriate depending on the context of the research [28, 29]. To accompany the matrix a rating and colour coding tool was developed by the authors to aid the assessment of the level of involvement described in each report (See Table 1). The rating and colour coding tool was tested on a selection of reports (n = 7) until agreement was reached between the authors.

**Table 1:** Rating and colour coding tool

Rating	Colour	Definition of analysis
Unmet	Red	No evidence of involvement
Partially met	Amber	Some evidence to suggest involvement
Fully met	Green	Clear Enough evidence of involvement Clear explanation and detail

*Step 2*: Analysis framework and rating tool.

We then created and iteratively tested an additional analysis framework (See Table 2) and rating tool (See Table 3) to assess the remaining NIHR reporting expectations. The Framework Method [30] which is commonly used for content analysis was used to organise data into a framework where rows represent cases and columns support codes. By using this method, we could compare vertically between NIHR expected reporting requirements (and GRIPP2 SF) and horizontally across each report.

**Table 2:** Analysis Framework

Item	Questions to support analysis
Aim*	(1) Did the authors describe the aim(s) of PPI in the study?
Methods	(2a) Is there a description of the methods used for involving CYP at different phases of the research process? This would include a description of: Level of involvement (e.g., consultation, collaboration, child-led) PPI population (e.g., numbers involved, age group, other demographics, and medical condition) Model of involvement (e.g., development of a Project Advisory Group, focus group, tapped into existing Young Person’s Advisory Group, etc.) (2b) Is there a description of the level of involvement within the various phases of the research process? This would include a description of: What phase of the research process are CYP involved? Is this involvement at just one phase of the research process or multiple levels?
Study results (outcomes)*	(3) Is there a description of the outcomes of PPI with CYP? (both positive and negative)
Impact	(4) Is there a description of the impact of PPI with CYP? (both positive and negative). This could include any description of the following impact: Impact on the study Impact on CYP Impact on researchers Impact on policy
Dissemination	(5) Is there a description of how CYP supported (or will support) the dissemination of research findings/results?
Reflections/critical perspectives*	(6) Is there a description of the conclusions and lessons learned from PPI with CYP?

*Only required when reporting against the GRIPP2SF

**Table 3:** Rating Tool

Rating	Code	Definition of analysis
Unmet	U	No evidence to address analysis questions
Partially met	P	Unclear Some evidence to address analysis questions Poor explanation and detail
Fully met	F	Clear Enough evidence to address analysis questions Clear explanation and detail

### Search

A search took place from October 2021 to February 2022, using the advanced search option within the NIHR Journals Library. Individual search terms (“children”, “child”, “young people”, “adolescents”, "infants”) were used to identify published paediatric focused reports, for any research type (primary research, evidence synthesis and methodology research), and any health category. Considering the implementation of the GRIPP2 guidance, the search parameters were reports published in 2018 up to 28th February 2022.

### Selection criteria

#### Inclusion criteria

Reports were included in the review if they met the inclusion criteria outlined in Table 4. Any interventional or observational study was included if the study population included CYP between the ages of 0–< 24 years, and the study involved this age group in any PPI activities. The age range was based on the World Health Organisation [31] definition of a ‘child’ as a person under the age of 18 years and a ‘young person’ as under the age of 24 years of age (10–24 years). Reports published in 2018 up to 28th February 2022.

**Table 4:** Inclusion and exclusion criteria

Inclusion criteria
Study population included children and young people between the age range of 0–< 24 years
PPI activities occurred with children and young people aged 0–< 24 years
Reports published in 2018 up to 28th February 2022
Any interventional or observational study
Exclusion criteria
Not relevant (study population didn’t include 0–< 24 years)
Reports waiting to be published
Reports that do not mention PPI
Reports that do not include children and young people in PPI activities
Unable to separate CYP population (as PPI advisors) from other populations (for example, parents or carers)
Insufficient information on PPI
Describes ‘engagement’ not involvement
Hard to distinguish between the actual research methods and PPI

#### Exclusion criteria

Exclusion criteria are summarised in Table 4. Reports were excluded if they didn’t include the study population, were still waiting to be published, did not mention PPI at all; did not include CYP in PPI reported activities, were unable to separate the CYP population (as PPI advisors) from other populations, described engagement rather than involvement activities, were hard to distinguish between qualitative research being undertaken and PPI activities specifically, and if there was insufficient information on PPI to assess according to the matrix.

### Selection procedure

Reports were independently screened by the lead author (JP) in two stages using title, abstract, and keywords initially, and then the full text manuscript. After removing duplicates, reports that were clearly irrelevant were excluded in the first stage of title and abstract review, and if the eligibility of the report was not clear, the full text was then reviewed in the second stage. The second stage included extracting any data that described PPI with CYP in the report, including footnotes, acknowledgements or links to peer reviewed journals that described PPI activities.

### Charting the data

A Microsoft Excel ^®^ version 2021 was developed to assist in extracting and analysing the information on CYP involvement within the published reports. Core data was extracted to characterise the cohort and to describe any CYP activity throughout the duration of the research study. Study characteristics included (a) funding category; (b) research type; (c) health category; (d) health research activity code; (e) age range of study cohort; (f) start date of study; (g) end date; (h) date study was published. Extracted data relating to PPI activities with CYP included (a) reported aims of PPI; (b) reported level of involvement within the various phases of the research process, and roles given (how CYP were involved, and what CYP did); (c) reported outcomes and impact; (d) reported dissemination plans; and (e) any reflections on lessons learnt (both positive and negative).

### Creating the tools to assess the comprehensiveness of reported involvement

(i) Assessing the level and opportunities offered to CYP
  Once the data were extracted, methods to judge the comprehensiveness of the description of the reported involvement activities were required. The matrix (Fig. 1) and accompanying rating tool (Table 2) was used to assess the reported level and opportunities offered to CYP across the different phases of the research process, as advised by the NIHR [32] (excluding the commissioning phase of research). Using the simplified typology of involvement identified by Lansdown (children are consulted, children collaborate with adult researchers, or child-led) we also added a fourth column that captured where CYP are not involved.
(ii) Co-design phase
  A small sub-group of healthcare professionals and a parent/PPI practitioner (co-authors GB, VH, ED) from the NIHR Paediatric Methodology Incubator (Patient and Public Involvement Working Group) was formed and led by the lead author (JP) to adapt and test the matrix, analysis framework, and accompanying assessment tools on the same seven reports used in step 1 of the methods. The sub-group met monthly for the duration of the project. Two additional workshops took place (one with five healthcare professionals from the wider team within the NIHR Paediatric Methodology Incubator, and one workshop with seven young people from the GenerationR Liverpool Young Person’s Advisory Group (YPAG)). Although the focus of both workshops was the same, each workshop adopted a slightly different approach. The healthcare professionals’ workshop (lasting 1 h 30 min) took place online. Participants were split into two groups, one facilitated by JP and the other facilitated by GB and VH. Each group received two PPI reports (of the seven selected reports used within the sub-group) in advance of the meeting accompanied by instructions on how to use the tools. We systematically captured and amended the wording of the matrix, analysis framework and accompanying tools considering feedback received during the workshop.

JP then facilitated a four-hour young person’s workshop which took place face-to-face. The seven young people included five boys (aged 13, 14, 16 (× 2), and 17 years old), and two girls (aged 17 and 18 years old). Each had been a member of the YPAG between 2 and 8 years. The workshop was designed to be interactive and was broken into two sessions and four activities, including a final discussion session (See the full YPAG Agenda in Additional file 1). The young people felt the matrix, analysis frameworks, and assessment tools were helpful to both reflect on how CYP are involved in research processes, and as helpful guides for research teams to adequately report PPI activities with CYP. The group did not suggest any further changes to the tools. Full details of these workshops will be presented in a separate paper.

Once the matrix, analysis framework and assessment tools were agreed the lead author then assigned these criteria independently for all reports that met the inclusion criteria. Further discussion was initiated with the sub-group if the lead author struggled to interpret the involvement description, for example if researchers were describing the qualitative aspect of the research, as opposed to describing how the involvement activities informed and supported the design of the qualitative methods.

NVivo 12 software was used to store, order and code data and to select supporting quotes. A rating was then assigned to each report and corresponding domain coding cell.

## Findings

### Search results

**Fig. 2:**
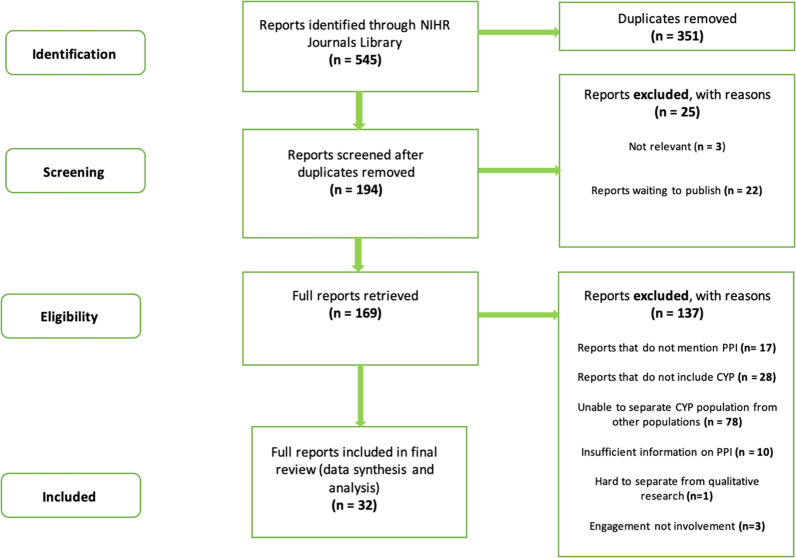
Schematic representation of report selection procedure

The initial search yielded 545 records, of which 351 were duplicates (see Fig. 2). Twenty-five reports were removed instantly as the study population was not relevant (n = 3), and n = 22 reports were waiting to be published. The search identified n = 11 reports published before April 2018; once assessed for eligibility, only four of these reports were included in the final data synthesis and analysis. In total 169 full reports were retrieved, of which 137 were excluded for the reasons identified in Fig. 2. In total, thirty-two reports were included in the final review.

### Characteristics of included reports

Details of the thirty-two reports are provided in Additional file 2. A summary is described below:

Twenty-seven of the thirty-two reports were primary research studies and five evidence synthesis studies. The primary research studies focused on the following health categories: cancer (n = 2); congenital disorders (n = 1); generic health relevance (n = 5); infection (n = 1); inflammatory and immune system (n = 1); mental health (n = 8); metabolic and endocrine (n = 2); musculoskeletal (n = 1); neurological (n = 1); oral and gastrointestinal (n = 2); reproductive health and childbirth (n = 3). The five evidence synthesis studies focused on mental health (n = 2) and generic health relevance (n = 3).

### How is PPI undertaken with children and young people?

The most common approach to involving CYP was through the medium of existing groups or networks, with 72% (23/32) choosing this approach. Of these 25, 60% (15/25) chose to access existing Young Person’s Advisory Groups (YPAGs) who have a remit to support the design and conduct of child health research [33–47], whilst 28% (8/25) reported some form of dialogue with existing youth forums such as, school advisory councils or care groups [36, 38, 39, 48–51], including one study accessed individuals through a PPI network within a Sexual and Reproductive Health Service (no description was provided within the report of who was involved in the network) [52]. 25% (8/32) established their own formal advisory group [53–60]. Of the remaining studies two set up focus groups or meetings with CYP [55, 60], two indicated they held one-off individual consultations with CYP [56, 61], two involved CYP in Study Steering Committees [53, 55], one reported involving young students in research training events held within a university [58], one reported involving CYP as mystery shoppers [52], and one report wasn’t clear on the chosen model [62].

### What opportunities are offered to children and young people?

**Fig. 3:**
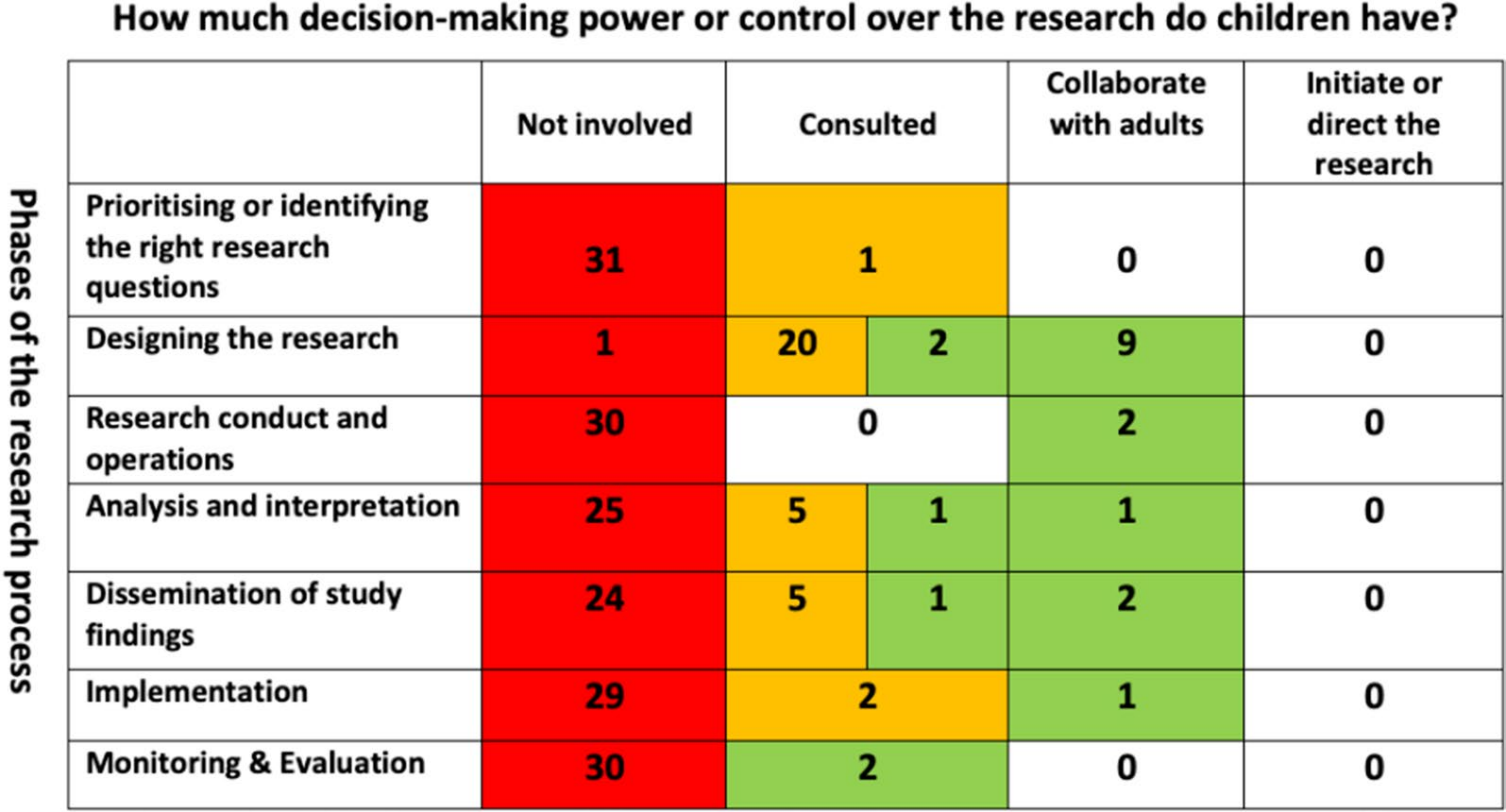
Final assessment of reported levels of CYP involvement

Although not all reports explicitly described the chosen level of CYP involvement or at the precise phase of research, using the adapted matrix (Fig. 1), and accompanying rating tool allowed us to assess the opportunities offered to CYP within each research project. This was also aided by a description of tasks associated to each phase of the research (See Additional file 3). Figure 3 summarises the final assessment of the reported levels of CYP involvement across the different phases of research highlighted in the reports.

At the phase of prioritising or* identifying the research question phase*: only one report [56] partially met the criteria for consulting CYP during this phase, and this was further evidenced in the published journal article separate from the main report [63]. The remaining thirty-one reports provided no evidence of involvement at this phase.

At the phase of* designing the research phase*: 97% of the reports (31/32) described varying levels of involvement in the design stage of the research. 71% of these reports (22/31) described involvement at the consultation level, with only 9% (2/22) fully meeting the criteria [38, 40], the remaining 91% (20/22) partially meeting the criteria [33, 34, 36, 37, 42–52, 54, 60–62, 64]. Instead, 29% fully met the criteria (9/31) describing a collaborative approach [39, 41, 53, 55–59, 65].

At the phase of* managing the research phase*: only 6% of the reports (2/32) highlighted a collaborative approach to involving CYP in the management of research, one via a young person being involved in the Study Steering Committee, [55] and one indicating direct linkage between a Children’s and Young Person’s Advisory Group to the Study Steering Committee [53].

At the phase of analysis and interpretation phase: 22% of the reports (7/32) indicated varying degrees of involvement in the analysis and interpretation phases of the research. Only one report fully met the criteria describing a collaborative approach to involving CYP in this phase [65]. The other (6/7) described consulting with CYP, five of which partially met the criteria [34–36, 46, 55], and one fully met the criteria [53].

At the phase of* dissemination of study findings phase*: 25% of the reports (8/32) described varying degrees of CYP involvement in the dissemination phase of the research. 62% of these reports (5/8) partially met the criteria that described a consultation approach to involving CYP at this phase [35, 36, 52, 57, 60]; one report fully met the criteria in the consultation category [56]; two reports fully met the criteria describing a collaborative approach [53, 65].

At the phase of* implementation phase*: 9% of the reports (3/32) indicated involvement in the implementation phase of the research. 67% of these reports (2/3) partially met the criteria at a consultation level [58, 65], and one fully met the criteria at a collaborative level [53].

At the phase of monitoring and evaluation phase: only 6% of the reports (2/32) fully met the criteria for involvement at this phase of the research, both at a consultation level [53, 65].

### Who is involved?

A full description of the demographics of CYP (gender, age, ethnicity, or health conditions versus healthy CYP) was weak for most reports. 84% (27/32) of the reports mentioned the age ranges of the CYP involved, the remaining five were less clear on the ages but indicated that primary school aged children were involved (n = 2), or secondary school pupils or teenagers were involved (n = 3). 96% (26/27) of the reports that mentioned age ranges implied the involvement of CYP between the ages of 7–25 years old. One report involved a six-year-old in a one-off exercise. It is not clear from the reports of the breakdown of ages for each activity how many younger children are involved compared to adolescents or young adults.

Only 9% of the reports (3/32) mentioned the gender breakdown of involved CYP [35, 39, 56]. Only 6% (2/32) of the reports mentioned the ethnicity of CYP [39, 56].

18% of the reports (6/32) referred to CYP having specific conditions or experiences including: mental health conditions (3/6) [50, 55, 65] such as, social anxiety disorder and experience of eating disorders; experience of having dental treatment (1/6) [62]; cancer (1/6) [56], and experience of appendicitis (1/6) [57]. 18% of the reports (6/32) addressed more general health experiences, such as the experience of living with long term conditions (2/6) [56, 65]; accessing health-care services who had experience of living with physical and/or developmental conditions relevant to the study (2/6) [41, 53], interested in medical research (1/6) [31], and one with experience of intensive care (1/6) [61].

### Frequency of involvement and types of activities

**Fig. 4:**
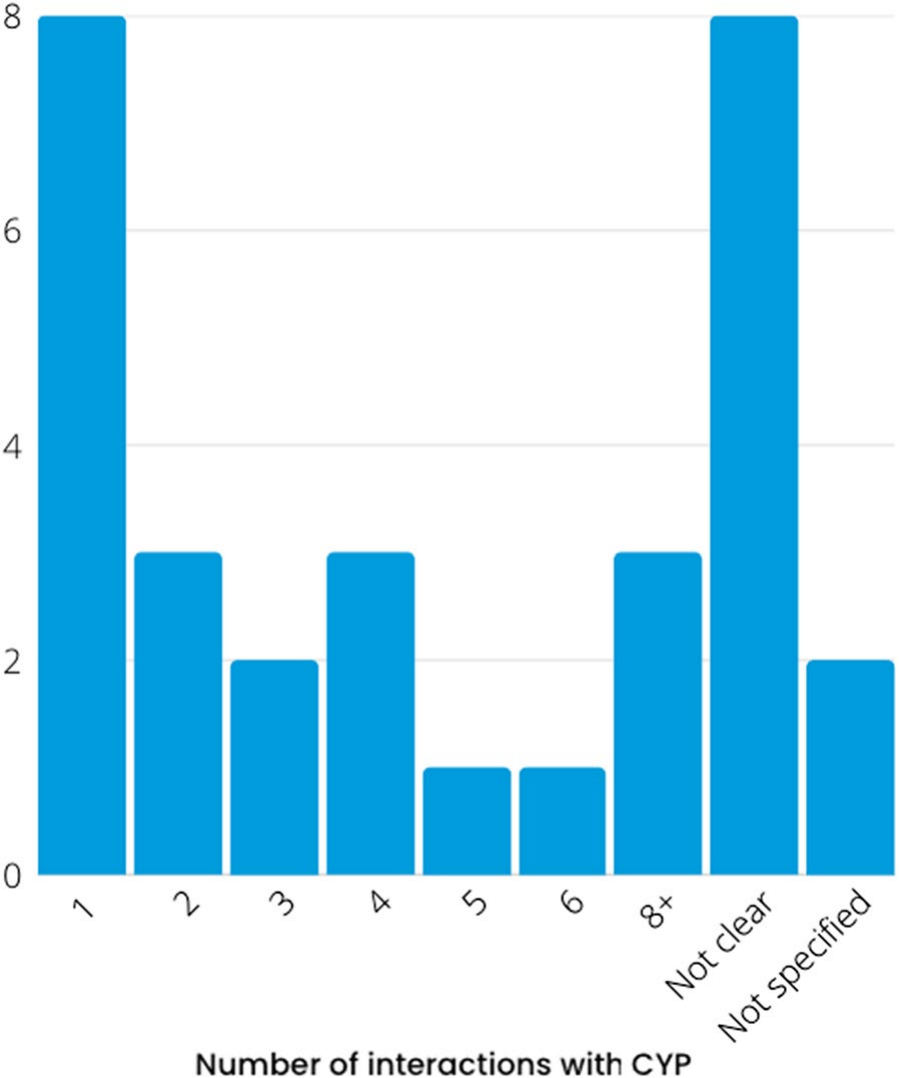
Frequency of interactions with CYP

The frequency of involvement (see Fig. 4) ranged from a one-off consultation up to nine consultations. One report indicated they met regularly with CYP (monthly) over the course of 5 years [53], and one report highlighted they held regular meetings (unknown amount) over the course of 10 years [56]. Both reports were large programme grants highlighting involvement in all work-packages and throughout the duration of the programme.

### Quality assessment of reports

The length of text to describe PPI with all stakeholders (parents, charities, adults, CYP, etc.) varied from one short paragraph to full chapters within the main report or appendices. Three reports had also published about their involvement with CYP in journal articles, separate from the main published report. 12% (4/32) reports provided detailed information but blurred lines between PPI activities with actual study methods, making it difficult to determine who was involved and how.

Only 12% of the reports fully (4/32) met all the criteria for reporting PPI against the NIHR reporting expectations (See Table 5), and all four reports also met all the GRIPP2SF criteria.

**Table 5:** Rating assigned to each report against NIHR reporting expectations

Report ID	Author	Aim*	(A) Methods (level & model)	(B) Methods (phase of research)	Outcomes*	Impact	Dissemination	Reflections*
NIHRJL01	Moore et al. [65]	F	F	F	F	F	F	F
NIHRJL02	Giles et al. [60]	U	P	F	F	F	P	P
NIHRJL03	Colver et al. [53]	F	F	F	F	F	F	F
NIHRJL04	Blair et al. [33]	U	P	P	U	U	U	U
NIHRJL05	Goodwin et al. [34]	U	P	P	F	F	U	U
NIHRJL06	King et al. [54]	U	P	P	P	P	U	U
NIHRJL07	Tancred et al. [35]	U	P	P	U	U	U	U
NIHRJL08	Ford et al. [48]	U	P	P	U	U	U	U
NIHRJL09	Bonell et al. [36]	U	P	F	U	U	U	U
NIHRJL10	Ramanan et al. [37]	U	P	P	U	U	U	U
NIHRJL11	Creswell et al. [55]	P	P	F	F	F	U	F
NIHRJL12	Mitchell et al. [64]	U	P	P	F	F	U	U
NIHRJL13	Ponsford et al. [38]	U	P	F	F	F	U	U
NIHRJL14	Alderson et al. [39]	U	F	F	F	F	U	U
NIHRJL15	Bray et al. [40]	F	P	F	F	F	U	P
NIHRJL16	Janssens et al. [49]	U	P	P	P	P	U	U
NIHRJL17	Maguire et al. [62]	U	P	P	P	U	U	U
NIHRJL18	Robling et al. [41]	F	F	F	F	F	F	F
NIHRJL19	Meiksin et al. [42]	U	P	P	P	U	U	U
NIHRJL20	Mallucci et al. [43]	U	P	P	U	U	U	U
NIHRJL21	Caldwell et al. [44]	F	P	P	P	U	U	U
NIHRJL22	Langton Hewer et al. [45]	U	P	P	U	U	U	U
NIHRJL23	Taylor et al. [56]	F	F	F	F	F	F	F
NIHRJL24	Hall et al. [57]	U	F	F	F	F	P	F
NIHRJL25	Cameron et al. [52]	U	P	P	P	U	U	U
NIHRJL26	Byford et al. [50]	U	P	P	U	U	U	U
NIHRJL27	Tume et al. [61]	F	P	P	P	U	U	U
NIHRJL28	Kidger et al. [46]	U	P	P	U	U	U	U
NIHRJL29	Adab et al. [51]	U	P	P	U	U	U	U
NIHRJL30	Bee et al. [58]	U	F	F	P	P	U	U
NIHRJL31	Cottrell et al. [47]	U	P	P	U	U	U	U
NIHRJL32	Griffiths et al. [59]	U	F	F	F	F	U	U

*Only required by GRIPP2SF

### Aim of research

Although the aim of PPI was not a NIHR mandatory field to complete, 22% of the reports (7/32) fully met this criterion [40, 41, 44, 53, 56, 61, 65] and one report partially met this criterion [55].

## Methods

- Level and model
  Only 25% (8/32) fully met the criteria for reporting the level and model of involvement [36, 41, 53, 56–59, 65]. The remaining reports partially met this criterion.
- Phase of research
  40% (13/32) fully met the criteria for reporting involvement at different phases of the research process [34, 36, 39–41, 53, 55–57, 59, 60, 64, 65]. The remaining reports partially met this criterion.

### Study results (outcomes)

41% of the reports (13/32) fully met the criteria describing the outcomes of PPI with CYP on study results [34, 38–41, 53, 55–57, 59, 60, 64, 65], 25% (8/32) partially met the criteria [45, 47, 49, 52, 54, 58, 61, 62], and the remaining 34% (11/32) did not meet the criteria. The most commonly reported outcomes focused on the positive impact on study design. These included: confirming the study importance, and study interventions and suitability of the research questions [56]; developing outcome measures important to CYP [56–58, 65], and core outcome set development [57]; contributing to the intervention development [64]; input into study documentation, including contributing to the design of interview topic guides and manuals aimed at CYP [39, 55, 59, 60], and survey development [56]; recruitment and retention materials, such as study information sheets or consent documentation, letters aimed at patients, videos, and advised on how to increase interest (of either PPI or research participation in the study) [38, 39, 41, 50, 52, 56, 59, 60, 62]; contributing to the interpretation and research findings [40, 59]; and subsequently dissemination to research participants [34, 38, 40, 52]; developing and testing website content [64]; interpreting study results and advising on implications of the study [38]; challenging research team’s understanding and assumptions [40]; providing input into evidence-based recommendations [58], and CYP assisted with the development of training materials for doctors [53].

### Impact

The NIHR defines impact as “*the changes, benefits and learning gained from the insights and experiences of patients, carers and the public when working in partnership with researchers and others involved in NIHR initiatives”* [66].

Based on this definition 41% of the reports (13/32) fully met the criteria describing the impact of PPI with CYP on the design of the study [34, 38–41, 53, 55–57, 59, 60, 64, 65]. Two of these reports [38, 65] provided helpful tables (see Tables 6 and 7) to map out PPI and perceived impact on the research process and intervention. 9% of the reports partially met (3/32) this criterion [49, 54, 58], and the remaining 50% (16/32) did not mention any impact.

**Table 6:** Example table used to describe end-user involvement

Activity	Date	Who?	End-user perspective represented	Impact
Planning stage				
Review methods stage				
Consultation stage				
Dissemination stage

**Table 7:**
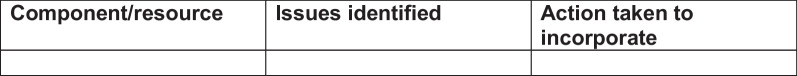
Example table used to highlight findings from PPI activities

Those that did not mention any impact focused on reporting (in varying degrees) the process of involvement and the roles CYP played within the project, without describing what difference the involvement made to the research, to CYP or to the researchers involved. For example, some reports mention CYP had helped to shape a patient information sheet but did not provide details of what happened as a result, or what changes the research team took on board. There was a lack of description on how CYP helped the design of the study, or how the young people felt about their involvement.

### Reported impacts of involvement on CYP

Only 6% of the reports (2/32) had undertaken some form of evaluation to measure the impact and experiences of CYP involvement [39, 53], and one report had undertaken a narrative self-reflection of the involvement experiences over the course of ten years [56]. All three reports published findings in separate journal articles [63, 67, 68].

Despite the lack of any formal evaluations of impact, the personal impacts on CYP were reported in19% of the reports (6/32) [39, 53, 55–57, 65], with only three including descriptions directly from CYP [39, 53, 65]. CYP felt that their involvement gave them the opportunity to meet other young people who shared the same or similar health conditions to them as one young person highlighted: “*It was good to hear other people’s point of view. I think it was really helpful having other people who have gone through the same things as you that understand you*”. They also felt that their experiences and contributions could make a real difference [65]. One young person wrote to the Principal Investigator of the study to highlight how her role had helped in her recovery from SAD [55]. One research teams reflections claimed that the benefits of being in a group gave young people the space to realise their cancer diagnosis which helped them to continue living their lives and gave young people the confidence to find their ‘new normal’ [56]. Reported impacts on CYP also included gaining skills such as research and technical skills [39, 56, 57], public speaking [56, 57] and confidence to interact with wider groups of young people and healthcare professionals [57, 65]. Impact on young people’s vocational or employability skills were highlighted in two reports [39, 55] such as recording achievements of involvement (by receiving certificates) and helping towards applying for university. Two reports [53, 55] highlighted how CYP felt valued and listened to adding to their self-confidence.

### Reported impacts on researchers

The reported impacts of involving CYP on researchers were only reported in 6% of the reports (2/32) [53, 57]. Both reports highlighted that involving CYP reinforced to research team members the importance of involving and gaining the views of CYP, ensuring that the research was of relevance to them. One report [53] simply felt that CYPs enthusiasm to be involved was motivating to team members, and one report [57] highlighted that it built their capacity to undertake effective PPI with CYP.

### Dissemination

12% of the reports (4/32) fully met the criteria describing CYP involvement in the dissemination activities of the study [41, 53, 56, 65], 6% partially met (2/32) this criterion [57, 60], and the remaining 81% (26/32) did not meet this criterion. Out of those who fully met the criteria two reports [41, 53] highlighted how CYP had contributed directly to dissemination activities throughout the programme beginning at the launch of the project, during the project (writing blogs of their experiences, presentation of emerging results, being interviewed for radio) running sessions at the final dissemination event and working with a theatre group to create an artistic interpretation of emerging results, having securing additional charity funding. One report [65] highlighted how CYP had recorded materials for Podcasts (one focused on the findings from the project and one to explain their experiences of being involved). One report [41] discussed how CYP activities had helped to refine the content and framing of the dissemination activities, in particular how study findings would be communicated to members of the public. Those who partially met the criteria alluded to some form of input from CYP to assist with the dissemination of study findings, explaining for example how CYP input will support the development of plain English summaries of the results, and focused on how findings will be distributed widely (e.g., via social media).

### Reflections and lessons learned

19% of the reports (6/32) fully met the criteria describing their reflections on PPI activities [41, 53, 55–57, 65], 6% partially met (2/32) this criterion [40, 60], the remaining 72% (24/32) did not meet this criterion.

### Reported challenges of involving CYP

Typically, the challenges of involving CYP in PPI were linked to practical and structural issues related to the respective study, such as population issues relating to recruitment and retention issues and sustaining CYP involvement over a long period of time, which required extensive relationship building [41, 56, 57]. Accommodating CYP’s availability (school commitments [60, 65], and capacity for schools to accommodate PPI requests [64] were also noted as key challenges. There was a need for more training for both PPI co-applicants and CYP [55], and recognition that explaining different methodologies to CYP can be difficult (e.g., involving young people in the design of patient information sheets was easier than explaining concepts such as core outcome set development). Structural constraints (research time frames, ethics procedures, allocated time, etc.) was noted as a key challenge [55, 56], and meeting CYP expectations particularly around the use of emerging technologies and social media which was constrained by resources and internal governance issues [56]. One report noted that if PPI was delivered by external PPI professionals independent of the research team meant that the research team were unable to discuss directly with the group which areas of feedback they were able to address, and those aspects of the study for whatever reason could not be modified [41].

### Reported enablers

Typically, some reports highlighted several solutions to tackle some of the challenges, including issues around recruitment and retention. For example, some teams highlighted the need for creating wide-reaching recruitment strategies, and having pre-existing relationships and networks to access was helpful (PPI networks, charity and patient organisation support, existing groups, connections in schools) [39, 41, 53, 55–57, 64]; communicating between meetings and maintaining regular contact [53, 56, 57], and valuing the input of CYP by thanking them on a regular basis [57] was deemed beneficial. Many reports focused on the logistics of PPI with CYP, for example making meeting spaces more welcoming, flexible, and enjoyable for CYP and allowing dedicated time to spend socialising with other members [53, 55–57, 65]. This also included having a dedicated PPI budget to offer payments, reimbursement of travel expenses, funds for refreshments, and having skilled PPI leads and PPI support to deliver the PPI activities with CYP [41, 55–57]. Having access to training and inductions for CYP was also deemed beneficial [40, 41] as was having clear role descriptions [40, 55]. It was also important to develop clear project specific payment policies [40, 55–57, 65], and other relevant safeguarding policies (i.e., out of hours, sickness policies) [56], and codes of conduct (e.g., alcohol, smoking and drug use) [56]. Finally, communication that is tailored to CYP capabilities about the research itself and PPI process including feedback was viewed as important to build trust and retain their involvement [41, 57].

## Discussion

This article provides an overview of reported PPI activities with CYP in 32 NIHR-funded study reports using a participation matrix, framework analysis and accompanying tools. Recording and reporting PPI is important, both to ensure transparency in relation to the contributions and roles of different stakeholders within the research process and to contribute to the evidence base within the field of PPI.

The assessment of the report’s highlights that the current reporting of involvement in health research is very poor, and rarely describes who was involved (demographic details of those involved, age, sex, ethnicity, etc.), and what outcomes and impact involvement had on the research process, on CYP and/or on researchers. The analysis framework and rating tool to assess the NIHR reporting requirements identified that despite the NIHR advice and guidance for authors to follow the GRIPP2 Checklist, only a small percentage followed this. We identified significant variability in the way PPI with CYP was reported. Common themes that emerged was that those who mentioned some form of dialogue with an existing YPAG or other existing forum (i.e., school group or PPI network), as opposed to establishing their own PPI structures, tended to report less and very rarely mentioned any outcomes or impact from that involvement. Some reports provided detailed information but either blurred the lines between PPI activities with actual study methods or focused the report on adult stakeholder involvement (i.e., parent/carer involvement, healthcare professional, teacher, etc.) as opposed to CYP involvement.

To better understand the opportunities and levels of CYP involvement throughout the research process we adapted an existing participation matrix that is more commonly applied to social science research/policy and practice rather than in a health research/PPI setting. The matrix and rating tool proved to be useful to explore the reported opportunities and levels of involvement of CYP across the different phases of the research process. Whilst using these to assess reported PPI activities was difficult due to the poor level of reporting, it did allow us to make some assessment of the opportunities offered to CYP across the phases of the research process. The matrix gave us a clearer picture of the reported opportunities offered to CYP, with the majority taking place during the design phase of the research, and only a small number of reports consulting and collaborating with CYP across other phases of the research. These reports tended to be long term studies (such as programme grants funded for five years and more) with dedicated PPI personnel and consumable budget to deliver PPI across the programme. We believe that despite the difficulties using the matrix, analysis framework and rating tools on published reports, they are extremely helpful tools to plan future activities with CYP (and other populations), and can ultimately support the quality, transparency, and consistency of the reporting process that the NIHR aspires to. We believe the tools can be helpful in a number of ways including: as supportive tools to be used from inception of the research idea to inform grant applications and help identify possible involvement levels/activity prior to funding; the tools follow the research pathway so research teams can truly understand the opportunities offered to CYP and the resources required to involve them in a meaningful way; they can also be used to co-develop the PPI plan with CYP so that CYP can choose the levels of involvement across the different phases of the research process. Furthermore, the tools could be used to reflect on PPI throughout the research project, recording when and how CYP contributed, what they influenced, what changed and why this mattered to either the research project, CYP and/or researchers.

Further work is required to understand why and how the NIHR, and other funders of health research (charities, UKRI, etc.) collect such information, and what their intentions are for sharing the findings with the wider research community to continuously improve best-practice in PPI.

### Strengths and Limitations

The current framework analysis had some limitations. First, limiting our search to reports submitted to the NIHR Journal Library, whilst ensuring a level of consistency, has limitations as searches in other journals or funders of different types of health research may have revealed different results. Second, the individual search terms used a large age range to identify paediatric studies which means that some reports may not have been included and this may have limited the findings. Despite these limitations, this review has strengths such as our commitment to CYP involvement, and the level of detail provided focusing on NIHR-funded studies only. The matrix, analysis framework and accompanying rating tools were deemed sufficient tools to assess the levels of involvement and opportunities offered to CYP, and assessment of how PPI with CYP described in the reports meet the NIHR reporting expectations.

## Conclusion

There is a paucity of knowledge regarding involvement activities in the research process in child health research. Questions surrounding PPI are now moving beyond the ‘why’ to the ‘how’, yet the reporting of PPI (in general) leaves knowledge users with an insufficient understanding of how the work was conducted, thus limiting its reproducibility, applicability, and impact. Furthermore, shared learning from previous experiences is lost and potentially a waste of resource that could otherwise be put to ensuring ‘meaningful’ involvement with CYP and other patient populations.

Reporting the processes and outputs of CYP involvement more rigorously may help child health researchers involve CYP more meaningfully by learning from the experiences, enablers and challenges faced by other researchers. Furthermore, sharing information about how CYP are involved in the research cycle via different activities can help researchers in planning and conducting future studies and to reflect on their current involvement practices. Such reporting of PPI with CYP should enhance the ability to develop evidence-based guidance around how to meaningfully involve CYP in paediatric health research, and to explore and evaluate the impact of their involvement. This knowledge may also help CYP gain more awareness about the ways they can contribute as ‘advisors’ or ‘co-researchers’ and the type of influence they can have. As other publications have highlighted [21–23] more needs to be done to seek the views of CYP involved in PPI activities and the impact being involved has on them as opposed to the impact on the research only.

## Supplementary Information

**Additional file 1:** YPAG Agenda.

**Additional file 2:** Summary of NIHR reports.

**Additional file 3:** Tasks associated to each phase of the research.

## Data Availability

The article is not drawing on data that is suitable to a data repository. Further information on how we facilitated the workshop meetings is available from the corresponding author.

## Footnotes

1 https://www.journalslibrary.nihr.ac.uk/about-us/
