# Supplementary material for: Reporting involvement activities with children and young people in paediatric research: a framework analysis
Source: Res Involv Engagem. 2023 Jul 31;9:61. doi: 10.1186/s40900-023-00477-8 (PMC10388467; doi:10.1186/s40900-023-00477-8)
Supplement: Supplementary file 1 — Additional file 1: YPAG Agenda. [file 40900_2023_477_MOESM1_ESM.docx]

**Supplementary file 1:** GenerationR YPAG Meeting Agenda

**Agenda**

**Session 1:** How can CYP get involved in research?

**Aim:** To help understand the level of CYP involvement at each phase of the research process.

**Time:** 1 hour

**Resources:** Introductory presentation slides, large heading showing the 7 phases of the research process, tasks linked to each phase of the research, large headings illustrating the different levels of involvement (children and not involved, consulted, collaborate with adult researchers, child-led), a bag of definitions for each level, handouts of NIHR reports, coloured dots and assessment criteria, pens and post-it notes.

**Activity 1:** Involvement in the research process

Instructions

- Explain how patients and the public are encouraged to be involved in each phase of the research process. Introduce each phase of the research to the group (see chart on the next page). The headings can be placed on a wall depending on the size of the room.
- Explain to the group that the activity will enable them to consider how children and young people can be involved in different phases of the research process. This will help them assess the reports.
- Split into small groups and give each group a pack of cards that describes possible tasks at each phase of the research process. In groups, they can decide where each task is placed (on a wall or a large piece of paper).
- Discuss the findings as a large group.

**Activity 2:** Levels of involvement.

Instructions

- Explore with the group what the different levels of involvement means to them (consultation, collaboration, etc).
- On a large sheet of paper (or wall) next to the phases of the research process, place the levels of involvement along the top. Each group will receive a bag of definitions to be placed on to the chart in the relevant column and row. Once complete discuss if it all makes sense, or if they would change anything.

**BREAK (30 minutes)**

**Activity 3:** Assessment of how authors describe the level of involvement.

Instructions

- In two groups read an example NIHR report (should only take 5 minutes)
- Using the matrix and analysis tool (printed as large handouts) place the relevant coloured dots on the chart.
- Discuss as a large group the findings and how the young people found the task, and whether using the coloured dots to aid analysis made sense and was helpful.

**LUNCH break (1 hour)**

**Session 2:** Review of NIHR reports

**Aim:** To assess how researchers report PPI with CYP.

**Time:** 1 hour 30 minutes

**Resources:** Instructions handout, assessment framework, coding rules, NIHR reports (x2), scorecard, pens and post-it notes.

**Activity 4:** Using the assessment framework and coding rules.

Instructions

- Each of the two groups will receive a copy of a PPI section of an NIHR report.
- Individually read the report and highlight any part of the report that mentions CYP involvement.
- Using the assessment framework and coding rules individually score the report and then discuss the findings in your group. Record any disagreements and key points and feedback to the larger group.
- Repeat the above tasks with a different report.
- Feedback to the wider group the scores and reflections about using the assessment framework and score card.

**Activity 5:** Discussion

Instructions

Discuss as a large group overall thoughts on the reports.

What did the group think about the matrix, analysis framework and assessment tools.

Was there anything missing that should be included?

Any final thoughts?

**FINISH**
