# Supplementary material for: Reporting involvement activities with children and young people in paediatric research: a framework analysis
Source: Res Involv Engagem. 2023 Jul 31;9:61. doi: 10.1186/s40900-023-00477-8 (PMC10388467; doi:10.1186/s40900-023-00477-8)
Supplement: Supplementary file 2 — Additional file 2: Summary of NIHR reports. [file 40900_2023_477_MOESM2_ESM.docx]

**Supplementary file 2:** Summary of NIHR reports

| **Report ID** | **Author** | **Title of report** | **Journal** | **Study population** | **Health category** | **Health research activity code** | **Research type** | **Actual start date** | **Anticipated or actual completion date** | **Date report published** |
| --- | --- | --- | --- | --- | --- | --- | --- | --- | --- | --- |
| NIHRJL01 | Moore et al (2019)^[65]^ | Interventions to improve the mental health of children and young people with long-term physical conditions: linked evidence syntheses | Health Technology Assessment | 0-25 years | Mental health | Individual care needs | Evidence synthesis | Jan-16 | Apr-17 | May-19 |
| NIHRJL02 | Giles et al (2019)^[60]^ | Brief alcohol intervention for risky drinking in young people aged 14–15 years in secondary schools:  the SIPS JR-HIGH RCT | Public Health Research | 14-15 years | Cancer | Primary prevention interventions to modify behaviours or promote well-being | Primary research | Sep-15 | Dec-17 | May-19 |
| NIHRJL03 | Colver et al (2019)^[53]^ | Facilitating the transition of young people with long-term conditions through health services from childhood to adulthood: the Transition research programme | Programme Grants for Applied Research | 10-24 years | Generic Health Relevance | Individual care needs | Primary research | May-12 | Not clear | May-19 |
| NIHRJL04 | Blair et al (2018)^[33]^ | Continuous subcutaneous insulin infusion versus multiple daily injections in children and young people at diagnosis of type 1 diabetes: the SCIPI RCT | Health Technology Assessment | 7mths-15 years | Metabolic and endocrine | Pharmaceuticals | Primary research | Jan-11 | Jun-14 | Aug-18 |
| NIHRJL05 | Goodwin et al (2018) ^[34]^ | Standing frames as part of postural management for children with spasticity. What is the acceptability of a trial to determine the efficacy of standing frames? | Health Technology Assessment | 1-18 years | Musculoskeletal | Individual care needs | Primary research | Oct-15 | Jul-17 | Sep-18 |
| NIHRJL06 | King et al (2019)^[54]^ | Sexual risk reduction interventions for patients attending sexual health clinics: a mixed-methods feasibility study | Health Technology Assessment | 16-25 years | Reproductive health and childbirth | Primary prevention interventions to modify behaviours or promote well-being | Primary research | Jun-15 | Aug-17 | Mar-19 |
| NIHRJL07 | Tancred et al (2019)^[35]^ | Interventions integrating health and academic education in schools to prevent substance misuse and violence: a systematic review | Public Health Research | 4-18 years | Generic Health Relevance | Primary prevention interventions to modify behaviours or promote well-being | Evidence synthesis | Jan-16 | Aug-17 | Sep-19 |
| NIHRJL08 | Ford et al (2019) ^[48]^ | Training teachers in classroom management to improve mental health in primary school children: the STARS cluster RCT | Public Health Research | 4-9 years | Mental health | Primary prevention interventions to modify behaviours or promote well-being | Primary research | Jun-12 | Jun-17 | Apr-19 |
| NIHRJL09 | Bonell et al (2019)^[36]^ | Modifying the secondary school environment to reduce bullying and aggression: the INCLUSIVE cluster RCT | Public Health Research | 11-15 years | Mental health | Primary prevention interventions to modify behaviours or promote well-being | Primary research | Mar-14 | Feb-18 | Nov-19 |
| NIHRJL10 | Ramanan et al (2019)^[37]^ | Adalimumab in combination with methotrexate for refractory uveitis associated with juvenile idiopathic arthritis: a RCT | Health Technology Assessment | 2-18 years | Inflammatory and immune system | Pharmaceuticals | Primary research | Not clear | Oct-15 | Apr-19 |
| NIHRJL11 | Creswell et al (2021)^[55]^ | Cognitive therapy compared with CBT for social anxiety disorder in adolescents: a feasibility study | Health Technology Assessment | 11-17.5 years | Mental health | Psychological and behavioural | Primary research | Mar-16 | Not clear | Mar-21 |
| NIHRJL12 | Mitchell et al (2020)^[64]^ | A peer-led intervention to promote sexual health in secondary schools: the STASH feasibility study | Public Health Research | 14-16 years | Infection | Primary prevention interventions to modify behaviours or promote well-being | Primary research | Jan-16 | Dec-18 | Nov-20 |
| NIHRJL13 | Ponsford et al (2021)^[38]^ | A school-based social-marketing intervention to promote sexual health in English secondary schools: the Positive Choices pilot cluster RCT | Public Health Research | 12-13 years | Reproductive health and childbirth | Primary prevention interventions to modify behaviours or promote well-being | Primary research | Apr-17 | Dec-19 | Jan-21 |
| NIHRJL14 | Alderson et al (2020)^[39]^ | Behaviour change interventions to reduce risky substance use and improve mental health in children in care: the SOLID three-arm feasibility RCT | Public Health Research | 12-20 years | Mental health | Individual care needs | Primary research | Mar-16 | Not clear | Sep-20 |
| NIHRJL15 | Bray et al (2020)^[40]^ | Powered mobility interventions for very young children with mobility limitations to aid participation and positive development: the EMPoWER evidence synthesis | Health Technology Assessment | 5 years+ | Generic Health Relevance | Medical devices | Evidence synthesis | May-18 | Jun-19 | Oct-20 |
| NIHRJL16 | Janssens et al (2020)^[49]^ | The transition from children’s services to adult services for young people with attention deficit hyperactivity disorder: the CATCh-uS mixed-methods study | Health and Social Care Delivery Research | 14-19 years | Mental health | Individual care needs | Primary research | Nov-15 | Aug-19 | Nov-20 |
| NIHRJL17 | Maguire et al (2020)^[62]^ | Best-practice prevention alone or with conventional or biological caries management for 3- to 7-year-olds: the FiCTION three-arm RCT | Health Technology Assessment | 3-7 years | Oral and Gastrointestinal | Surgery | Primary research | Apr-10 | Dec-17 | Jan-20 |
| NIHRJL18 | Robling et al (2021)^[41]^ | The Family Nurse Partnership to reduce maltreatment and improve child health and development in young children: the BB:2–6 routine data-linkage follow-up to earlier RCT | Public Health Research | 16-19 years | Generic Health Relevance | Primary prevention interventions to modify behaviours or promote well-being | Primary research | Feb-14 | Not clear | Feb-21 |
| NIHRJL19 | Meiksin et al (2020)^[42]^ | A school intervention for 13- to 15-year-olds to prevent dating and relationship violence: the Project Respect pilot cluster RCT | Public Health Research | 13-15 years | Generic Health Relevance | Primary prevention interventions to modify behaviours or promote well-being | Primary research | Mar-17 | Dec-19 | Apr-20 |
| NIHRJL20 | Mallucci et al (2020)^[43]^ | Silver- impregnated, antibiotic-impregnated or non-impregnated ventriculoperitoneal shunts to prevent shunt infection" the BASICS three-arm RCT | Health Technology Assessment | Neonates-91 years | Neurological | Medical devices | Primary research | Mar-13 | Feb-19 | Apr-20 |
| NIHRJL21 | Caldwell et al (2021)^[44]^ | School-based interventions to prevent anxiety, depression and conduct disorder in children and young people: a systematic review and network meta-analysis | Public Health Research | 4-19 years | Mental health | Primary prevention interventions to modify behaviours or promote well-being | Evidence synthesis | Oct-16 | Nov-18 | Aug-21 |
| NIHRJL22 | Langton Hewer et al (2021)^[45]^ | Intravenous or oral antibiotic treatment in adults and children with cystic fibrosis and Pseudomonas aeruginosa infection: the TORPEDO-CF RCT | Health Technology Assessment | 28 days+ (no upper age limit) | Congenital Disorders | Pharmaceuticals | Primary research | Dec-09 | Not clear | Nov-21 |
| NIHRJL23 | Taylor et al (2021)^[56]^ | Specialist cancer services for teenagers and young adults in England: BRIGHTLIGHT research programme | Programme Grants for Applied Research | 13-24 years | Cancer | Organisation and delivery of services | Primary research | Dec-11 | Not clear | Nov-21 |
| NIHRJL24 | Hall et al (2021)^[57]^ | Conservative treatment for uncomplicated appendicitis in children: the CONTRACT feasibility study, including feasibility RCT | Health Technology Assessment | 4-15 years | Oral and Gastrointestinal | Management and decision making | Primary research | Jul-16 | Oct-18 | Feb-21 |
| NIHRJL25 | Cameron et al (2021)^[52]^ | Provision of the progestogen-only pill by community pharmacies as bridging contraception for women receiving emergency contraception:  the Bridge-it RCT | Health Technology Assessment | 16+ years | Reproductive Health and Childbirth | Organisation and delivery of services | Primary research | Not clear | Not clear | May-21 |
| NIHRJL26 | Byford et al (2019)^[50]^ | Alternative community-based models of care for young people with anorexia nervosa: the CostED national surveillance study | Health and Social Care Delivery Research | 8-17 years | Mental health | Organisation and delivery of services | Primary research | Jun-13 | Not clear | Oct-19 |
| NIHRJL27 | Tume et al (2020)^[61]^ | Routine Gastric Residual Volume measurement to guide enteral feeding in mechanically ventilated Infants and Children: the GASTRIC Feasibility Study | Health Technology Assessment | Birth to 16 years | Generic Health Relevance | Primary prevention interventions to modify behaviours or promote well-being | Primary research | Apr-18 | Not clear | May-20 |
| NIHRJL28 | Kidger et al (2021)^[46]^ | Mental health support and training to improve secondary school teachers’ well-being: the WISE cluster RCT | Public Health Research | Students in year 8 and 10, and teachers | Mental health | Primary prevention interventions to modify behaviours or promote well-being | Primary research | Dec-15 | Not clear | Nov-21 |
| NIHRJL29 | Adab et al (2018)^[51]^ | The West Midlands ActiVe lifestyle and healthy Eating in School children (WAVES) study: a cluster randomised controlled trial testing the clinical effectiveness and cost-effectiveness of a multifaceted obesity prevention intervention programme targeted at children aged 6-7 years | Health Technology Assessment | Primary school children | Metabolic and Endocrine | Primary prevention interventions to modify behaviours or promote well-being | Primary research | Nov-10 | May-16 | Feb-18 |
| NIHRJL30 | Bee et al (2018)^[58]^ | A rapid evidence synthesis of outcomes and care utilisation following self-care support for children and adolescents with long term conditions (REFOCUS): reducing care utilisation without compromising health outcomes | Health and Social Care Delivery Research | 0-18 years | Generic Health Relevance | Organisation and delivery of services | Evidence synthesis | May-15 | Oct-16 | Jan-18 |
| NIHRJL31 | Cottrell et al (2018)^[47]^ | A pragmatic randomised controlled trial and economic evaluation of family therapy vs. treatment as usual for young people as seen after second or subsequent episodes of self-harm: the self-harm intervention – family therapy (SHIFT) trial | Health Technology Assessment | 11-17 years | Mental health | No category | Primary research | Not clear | Not clear | Mar-18 |
| NIHRJL32 | Griffiths et al (2018)^[59]^ | The role of digital communication in patient-clinician communication for NHS providers of specialist clinical services for young people (the long-term conditions young people networked communication (LYNC) study): a mixed -methods study | Health and Social Care Delivery Research | 16-24 years | Generic Health Relevance | Individual care needs; Organisational and delivery of services | Primary research | May-14 | Aug-16 | Feb-18 |
