# Supplementary material for: Reporting involvement activities with children and young people in paediatric research: a framework analysis
Source: Res Involv Engagem. 2023 Jul 31;9:61. doi: 10.1186/s40900-023-00477-8 (PMC10388467; doi:10.1186/s40900-023-00477-8)
Supplement: Supplementary file 3 — Additional file 3: Tasks associated to each phase of the research. [file 40900_2023_477_MOESM3_ESM.docx]

**Supplementary file 3:** Tasks associated to each phase of the research

| Phase of research | Description and why it is important | Could include the following tasks |
| --- | --- | --- |
| Prioritising or identifying the right research questions | Involvement in the prioritisation of research topics and questions to ensure that they are aligned with CYP who have a personal stake in the research. | - Inform research topics and priorities important to them. This could be via informal focus group discussions and questionnaires, through to more formal and structured processes, such as a James Lind Alliance Priority Setting Partnerships - Inform the development of the research question.^[[1]](#footnote-1)^ |
| Designing the research | Involvement in study design to make the research more accessible, relevant and ethical. | - Clarifying the research question and inform its importance. - Ensuring the methods selected are appropriate for patients. - Reviewing and commenting on proposed questionnaires and data collection methods. - Helping to ensure that the research proposed and chosen methods are ethical. - Assisting in writing the patient information and/or assent/consent forms. - Review and comment on proposed questionnaires and data collection methods. - Informing areas where patients and the public could be involved. - Defining outcome measures. - Raising awareness about costs of involvement, expenses, and prompt researchers to cost for involvement. - Advising on the appropriateness of the Lay Summary. |
| Research conduct and operations | Involvement in the management and conduct of studies to maintain a patient/public perspective throughout an individual project or a programme of work. | - Steering the project throughout the research process (e.g., named as co-applicants or peer researchers). - Supporting recruitment to the study (e.g designing recruitment posters or adverts, identifying suitable methods to reach CYP, sharing surveys with their peers, etc). - Designing the detailed protocol. - Supporting the interview/focus group processes (e.g., developing topic guides, co-facilitating interviews/focus groups etc). |
| Analysis and interpretation | Involvement in analysis and interpreting research findings helps to identify themes that may be overlooked by researchers, checks the validity of the conclusions from a CYP perspective and highlight findings that are more relevant to patients and/or public. | - Assisting the research team in developing themes from data. - Identifying and refining themes from qualitative data - Commenting on quantitative/qualitative analysis and how to portray these findings to CYP - Helping to draw conclusions to be used in future research. |
| Dissemination of study findings | Involvement in the dissemination of study findings can ensure that the findings are widely disseminated so they can influence and change practice for the better. | - Advising on different avenues for disseminating results. - Producing research updates that are patient/public friendly. - Jointly presenting the findings with researchers. - Writing information for local patient groups/hospitals etc (press releases, website blogs, social media posts, etc). - Assisting in getting results/findings published on charities/voluntary organisations websites. - Producing summaries of findings. - Co-authoring journal publications or reports. |
| Implementation | Involvement in the implementation of findings can influence, support, and add strength to the way research is taken into practice. | - Develop patient information materials for new services/interventions within hospitals, GP surgeries etc. - Build relationships with key agencies and speak with policy makers. |
| Monitoring & Evaluation | Involvement in monitoring and evaluation of CYP activities helps to build an evidence base of what worked well and what didn’t, and the impact of involvement on the research. | - Involved in evaluating the process of involvement (e.g., given the space to reflect on their role and what they have learned, and the impact on the research project). |

1. Depending on the type of funding call, topics might have already been decided by the research funder or commissioner. Members of the public might have been involved in the identification and prioritisation of the topics by the research funding organisation. See briefing notes for researchers <https://www.nihr.ac.uk/documents/briefing-notes-for-researchers-public-involvement-in-nhs-health-and-social-care-research/27371?pr=#Identifying_and_prioritising_research> [↑](#footnote-ref-1)
